# MarkerDB: an online database of molecular biomarkers

**DOI:** 10.1093/nar/gkaa1067

**Published:** 2020-11-27

**Authors:** David S Wishart, Brendan Bartok, Eponine Oler, Kevin Y H Liang, Zachary Budinski, Mark Berjanskii, AnChi Guo, Xuan Cao, Michael Wilson

**Affiliations:** Department of Biological Sciences, University of Alberta, Edmonton, AB T6G 2E9, Canada; Department of Computing Science, University of Alberta, Edmonton, AB T6G 2E8, Canada; Department of Laboratory Medicine and Pathology, University of Alberta, Edmonton, AB T6G 2B7, Canada; Faculty of Pharmacy and Pharmaceutical Sciences, University of Alberta, Edmonton, AB T6G 2H7, Canada; Department of Biological Sciences, University of Alberta, Edmonton, AB T6G 2E9, Canada; Department of Biological Sciences, University of Alberta, Edmonton, AB T6G 2E9, Canada; Department of Biological Sciences, University of Alberta, Edmonton, AB T6G 2E9, Canada; Department of Biological Sciences, University of Alberta, Edmonton, AB T6G 2E9, Canada; Department of Biological Sciences, University of Alberta, Edmonton, AB T6G 2E9, Canada; Department of Biological Sciences, University of Alberta, Edmonton, AB T6G 2E9, Canada; Department of Biological Sciences, University of Alberta, Edmonton, AB T6G 2E9, Canada; Department of Biological Sciences, University of Alberta, Edmonton, AB T6G 2E9, Canada; OMx Personal Health Analytics, Inc., 406–10158 103 St NW, Edmonton, AB T5J 0X6, Canada

## Abstract

MarkerDB is a freely available electronic database that attempts to consolidate information on all known clinical and a selected set of pre-clinical molecular biomarkers into a single resource. The database includes four major types of molecular biomarkers (chemical, protein, DNA [genetic] and karyotypic) and four biomarker categories (diagnostic, predictive, prognostic and exposure). MarkerDB provides information such as: biomarker names and synonyms, associated conditions or pathologies, detailed disease descriptions, detailed biomarker descriptions, biomarker specificity, sensitivity and ROC curves, standard reference values (for protein and chemical markers), variants (for SNP or genetic markers), sequence information (for genetic and protein markers), molecular structures (for protein and chemical markers), tissue or biofluid sources (for protein and chemical markers), chromosomal location and structure (for genetic and karyotype markers), clinical approval status and relevant literature references. Users can browse the data by conditions, condition categories, biomarker types, biomarker categories or search by sequence similarity through the advanced search function. Currently, the database contains 142 protein biomarkers, 1089 chemical biomarkers, 154 karyotype biomarkers and 26 374 genetic markers. These are categorized into 25 560 diagnostic biomarkers, 102 prognostic biomarkers, 265 exposure biomarkers and 6746 predictive biomarkers or biomarker panels. Collectively, these markers can be used to detect, monitor or predict 670 specific human conditions which are grouped into 27 broad condition categories. MarkerDB is available at https://markerdb.ca.

## INTRODUCTION

Biomarkers are the signposts of biology. Just as road signs tell drivers what's ahead or what's happening on the road, biomarkers provide scientists with the same kind of information – but for biological systems. Formally, a biomarker is a measurable substance or characteristic in an organism that is indicative of some phenomenon such as a condition, a disease, a diet, an intervention, or an environmental exposure. In this regard, biomarkers can be categorized into three broad types: (i) molecular (chemicals, proteins or genes), (ii) cellular (cell type, cell morphology, tissue histology) or (iii) imaging (X-ray, CT, PET or MRI features). The choice of a biomarker type very much depends on the phenomenon being studied and the biological system under investigation. As a result, a diverse variety of biomarkers are used in many different disciplines ranging from medicine, to nutritional science, to environmental monitoring, to drug testing and toxicology, even to soil science.

In medicine, the primary purpose of biomarkers is to diagnose, prognose or predict disease. They can also be used to assess exposures to drugs, toxins, pollutants, foods or other ingested substances. Interest in biomarkers, especially molecular biomarkers, has grown considerably over the past 50 years. In 1969, there were fewer than 100 papers published on biomarkers, by 2019 there >67 000. In total, >1 000 000 papers with mentions of the words ‘biomarker’ or ‘biomarkers’ are listed in PubMed. This rapid growth in biomarker research mirrors the interest in biomarkers by industry. Biomarkers are big business. The global market for medical biomarkers is worth >$50 billion/year and growing at a rate of >13% a year (https://www.grandviewresearch.com/industry-analysis/biomarkers-industry). This growth partly driven by the fact that biomarkers (i.e. molecular biomarkers) are touted as foundational to both precision medicine (1) and molecular medicine (2).

Given the importance of molecular biomarkers and given the large number of known biomedical biomarkers it is surprising to find that there are actually very few molecular biomarker databases. Those that are available are typically subscription-based, commercial databases such as the Global Online Biomarker Database – GOBIOM (https://gobiomdbplus.com/), Biomarkerbase (https://www.biomarkerbase.com/) or the Human Gene Mutation Database (http://www.hgmd.cf.ac.uk/ac/index.php). While these are excellent, comprehensive resources, they do not provide any cross-database interconnectivity and they obviously do not adhere to FAIR database principles (3). Furthermore, they are somewhat limited in their coverage (no karyotype biomarkers, little in terms of chemical biomarkers, no SNP biomarker data, etc.) and information content (no data on biomarker cut-off values, sensitivity, specificity, receiver-operating characteristic [ROC] curves, disease/condition descriptions, etc.). In addition to these commercial tools, there are a small number of very specialized, open-access molecular biomarker databases such as OncoMX (4), which specializes in cancer biomarkers, the colorectal cancer biomarker database or CBD (5), which focuses on colorectal cancer biomarkers, ResMarkerDB (6), which specializes in therapeutic response biomarkers and the urinary protein biomarker database or UPBD (7), which is limited to urinary protein biomarkers. Most of the biomarkers described in these databases are very specific (protein only, gene only), limited to investigational or pre-clinical biomarkers and provide relatively little information about the molecular marker itself or the specific condition/disease associated with those biomarkers.

Over the past decade our laboratory has developed a number of databases that contain a significant amount of medical biomarker data, but primarily as incidental information. These include HMDB (8) which contains chemical biomarker data on inborn errors of metabolism, Exposome-Explorer (9), which contains dietary and pollution exposure biomarker data and GWAS-ROCS (10), which contains SNP-disease biomarker data. Unfortunately, these data are not ideally formatted for molecular biomarker queries. Furthermore, they do not typically include critical information, such as biomarker cut-off values, sensitivity, specificity or disease/condition descriptions, needed to make them truly useful to the medical or clinical research community. Overall, the state of open access molecular biomarker resources is rather abysmal with biomarker data scattered incoherently and incompletely across many specialized databases. This makes it almost impossible for scientists or clinicians to find what molecular biomarkers are known for a given disease, what biomarkers are approved for clinical use, which biomarkers or biomarker tests are most useful, or whether it is even worthwhile to pursue finding a new biomarker.

Ideally what is needed is an open-access, comprehensive biomarker database that covers a wide range of molecular biomarkers for a broad range of biomedical applications. This would allow scientists and clinicians to quickly identify, compare and assess known molecular biomarkers for specific conditions and make informed decisions about appropriate clinical actions or research directions. Such a database should cover chemical, genetic (DNA mutation, DNA SNP, karyotypic) and protein biomarkers. It should also capture both clinically approved as well as investigational (or pre-clinical) biomarkers including diagnostic, predictive and prognostic biomarkers. The data contained in such a resource should cover diverse diseases (genetic, cancer, infectious, metabolic, environmental), conditions (phenotypes or health states) and exposures (drugs, food, pollutants). Furthermore, the data in such a database should include rich, detailed descriptions about the markers themselves, about the diseases/conditions with which they are associated and quantitative information about the biomarker performance (cut-off values, sensitivity, specificity, ROC curves, reference values and references).

Here we wish to describe just such a database—called MarkerDB (https://markerdb.ca). MarkerDB is a comprehensive, richly annotated database of molecular biomarkers that covers both clinically approved and pre-clinical/investigational markers described in the literature. It includes four major types of molecular biomarkers (chemical, protein, DNA and karyotypic) and four biomarker categories (diagnostic, predictive, prognostic and exposure) which are associated more than 27 broad disease categories and over 600 different conditions or diseases. MarkerDB is designed to be easily navigable and allows users to browse, query or search by disease or conditions, by biomarker names/identifiers, by sequence (gene or protein) or by chemical structure (SMILES or InChI). Each biomarker and each disease/condition is richly annotated with images, hyperlinks and references. Likewise, quantitative data on the biomarker (or biomarker panel) performance is also provided including cut-off values, sensitivity, specificity, ROC curves, reference values and references. Indeed, a central goal of MarkerDB is to include detailed biomarker performance data for as many markers as possible so that the quality and utility of a biomarker or biomarker panel can be objectively evaluated. A more complete description of MarkerDB, its design, its content, its sources and its curation process is given below.

## MarkerDB DESCRIPTION AND CONTENT

MarkerDB (version 1.0) contains >27 759 molecular biomarkers covering 26 493 clinically approved markers and 1226 pre-clinical or investigative biomarkers. The vast majority (95.6% or 26 628 markers) are singular markers (one condition-one marker) while a minority (4.4% or 1219 markers) a part of MarkerDB’s collection of 451 multi-marker panels. The biomarkers in MarkerDB are partitioned into four molecular categories including 142 protein biomarkers, 1089 chemical biomarkers, 154 karyotype biomarkers and 26 374 DNA biomarkers. The DNA biomarkers are further classified into 26 021 DNA mutations associated with 760 human genes, 353 SNPs associated with 178 human genes, and 23 microbial/viral genes associated with 16 infectious microbes and viruses. The molecular markers in MarkerDB are further grouped into 25 560 diagnostic biomarkers, 102 prognostic biomarkers, 265 exposure biomarkers and 6746 predictive biomarkers (or biomarker panels). Collectively, these markers can be used to detect, monitor or predict 699 specific human conditions/diseases which are classified into 27 broad condition categories. The entire database occupies 328 Mb of data. Table 1 provides additional detailed statistics regarding the content of MarkerDB.

**Table 1.** Detailed data content of MarkerDB (Version 1.0)

Biomarker data in MarkerDB	Total
Total number of biomarkers	27 759
Number of clinically approved biomarkers	26 493
Number of pre-clinical or investigational biomarkers	1226
Number of singular biomarkers (one marker – one condition)	26 628
Number of biomarkers in multi-marker panels	1219
Number of multi-marker panels	451
Number of chemical biomarkers	1089
Number of protein biomarkers	142
Number of DNA mutation biomarkers	26 021
Number of DNA SNP biomarkers	353
Number of viral/bacterial DNA biomarkers	23
Number of karyotype biomarkers	154
Number of diagnostic biomarkers	25 560
Number of prognostic biomarkers	102
Number of predictive biomarkers	6746
Number of Exposure (diet and chemical) biomarkers	265
Number of diet-related biomarkers	49
Number of diseases/conditions with biomarkers	699
Average number of biomarkers per disease	41
Percentage of chemical biomarkers with ROC or cut-off data	98%
Percentage of protein biomarkers with ROC or cut-off data	29%
Percentage of DNA biomarkers with ROC or cut-off data	>90%
Number of chemicals with structures	978
Number of proteins with 3D structures/PDB links	121
Number of protein sequences	142
Number of DNA sequences	26 374
Average number of words in disease descriptions	147
Average number of words in chemical descriptions	214
Average number of words in protein descriptions	176
Average number of words in gene descriptions	89
Number of references (PubMed IDs or DOIs)	5391

The vast majority of information in this first version of MarkerDB was collected, illustrated or annotated from original source data or primary literature data by a team of curators over the course of nearly 10 years. Data from our own databases or other online resources were also used to supplement or inform the curation process. A key challenge with extracting data from many biomarker papers, biomarker databases or biomarker reference publications is the generally poor quality of reporting on biomarker performance (11). Key to assessing a biomarker's performance is its sensitivity, specificity, ROC curve, reproducibility, statistical significance, and the availability of threshold or cut-off values. Most biomarker papers and textbooks do not include this information, meaning that most of the published investigational markers (and even some clinically used markers) did not meet the minimum standards for inclusion in MarkerDB. In some cases, it is possible to extract or re-generate this biomarker performance data using the information provided in the paper or by supplementing it with additional information from standard reference tables or online reference databases (this was done for many chemical and SNP biomarkers). Following is a more detailed description of the content, source and data curation process for each of the five major molecular marker categories in MarkerDB.

### Chemical biomarkers

The chemical biomarkers in MarkerDB include markers for a variety of inborn-errors of metabolism or genetic conditions, cancers, metabolic disorders, infectious diseases, drug exposures, chemical/pollutant exposures and dietary intake. In total, the 1089 chemical markers in MarkerDB are associated with 448 diseases or conditions and 106 exposures. Altogether, 336 (31%) of these chemical biomarkers are clinically approved (in various jurisdictions) or are part of laboratory developed tests (LDTs) while 753 (69%) are classified as investigative, research-use only or pre-clinical. One of the inherent strengths of chemical biomarkers (and clinical chemistry in general) is that the vast majority of chemical biomarkers can be absolutely, quantitatively measured with high precision and accuracy. This ensures that they can be widely used and applied to many different populations using a range of measurement platforms. A key source for much of the clinical chemistry data for the disease-compound associations of ‘singular’ biomarkers in MarkerDB was our own HMDB (8). However, the HMDB data required additional reviewing, upgrading, annotation and original literature searching to meet the high data standards of MarkerDB. Some of the more tedious and more easily ‘calculable’ annotation was facilitated by two in-house chemical annotation tools called DataWrangler and ChemoSummarizer (12). Currently, every chemical marker in MarkerDB has one or more disease associations along with a MarkerDB accession number, structure, a compound description, names/synonyms, physicochemical data, disease concentration(s), normal concentration(s), gender, age range (adult/child/newborn), biofluid (that the marker is normally measured in), a ROC curve, sensitivity, specificity, significance (*P*-value), one or more literature references and external hyperlinks to other online databases (OMIM, GenBank, UniProt, HMDB, PubMed, PDB, etc.). If cut-off values, reference ranges, ROC curves and/or sensitivity/specificity data were provided in the original literature source, these were used as provided and cited. If no specific data on biomarker performance was available, HMDB-derived age-specific or gender-specific concentration ranges and literature-derived disease-specific values were used to calculate appropriate cut-off values (to maximize AUROC), as well as the appropriate reference ranges and sensitivity/specificity data using standard protocols (11). A similar protocol was followed for multi-marker chemical panels, wherein only those papers with absolute quantification and sufficient information on the biomarker set (cut-off values, reference ranges and sensitivity/specificity data, ROC data) or calculable information that could be re-generated from the published data were used (10,11).

For compiling MarkerDB data on dietary, drug and chemical exposure biomarkers, information from HMDB, Exposome-Explorer (9) and NHANES (National Health and Nutrition Examination Survey) (13) was used. The same process of primary literature review, data assessment/extraction and, if necessary, data regeneration was undertaken. Again, the availability of fully quantitative chemical data greatly facilitated the annotation and data generation process.

### Protein biomarkers

The 142 protein biomarkers in MarkerDB cover >160 diseases. A significant majority (87%) of these protein biomarkers are clinically approved while a much smaller proportion (13%) are classified as investigative, research-use only or pre-clinical. Almost all protein markers are detected and quantified via enzyme-linked immune-sorbet assays (ELISAs). The reliance on antibody assays makes protein quantification inherently less reliable and less universal than chemical quantification. As a result, unlike with chemical biomarkers, no attempt was made to independently calculate or regenerate protein biomarker data from ‘partial’ or incomplete protein biomarker data in published papers. All of the protein biomarker data in MarkerDB was extracted from the primary literature with sources such as OncoMX (5), CBD (6), and UPBD (7) providing useful reference leads. The annotation of protein biomarkers was done manually and facilitated by an in-house protein annotation tool called BioSummarizer (12). Protein structure data was collected manually from the Protein DataBank – PDB (14). Currently, all protein markers in MarkerDB have one or more disease associations along with a MarkerDB accession number, a sequence, a structure (if available), a detailed protein description, protein names/synonyms, protein physicochemical data, disease concentration(s), normal concentration(s), gender, age range (adult/child/newborn), biofluid (that the marker is normally measured in), one or more literature references and external hyperlinks to other online databases. Biomarker performance data, such as cut-off values, a ROC curve, sensitivity, specificity, significance (*P*-value), etc. are available for 30% of the protein biomarkers. All biomarker performance data were directly taken from the original literature source and cited. This was done for both singular protein markers and multi-marker panels.

### DNA biomarkers

DNA biomarkers make up the largest single category of markers in MarkerDB with 26 374 markers associated with >319 diseases/conditions. A significant majority (98%) of these DNA biomarkers are clinically approved while a much smaller proportion (2%) are classified as investigative or research-use only. DNA biomarkers within MarkerDB are further classified into 26 021 DNA mutation markers associated with 209 diseases, 353 SNP markers associated with 70 conditions or diseases, and 23 microbial/viral genes associated with 16 infectious diseases. The main source of DNA mutation data (and clinically approved mutation tests) was from the Genetic Testing Registry (15) with sequence data extracted from GenBank (16) and disease information extracted from OMIM (17) or the Genetics Home Reference (https://ghr.nlm.nih.gov/). The main source for DNA SNP biomarkers was from our own database, GWAS-ROCS (10), with disease information extracted from OMIM (17) or the Genetics Home Reference (https://ghr.nlm.nih.gov/). The remaining data on microbial/viral biomarker genes were obtained entirely through primary literature or patent searches. The annotation of all DNA biomarkers was done manually and facilitated by an in-house gene/protein annotation tool called BioSummarizer (12). Currently, all DNA markers in MarkerDB have one or more disease associations along with a MarkerDB accession number, a sequence (with disease variants marked), a detailed gene description (if in a coding region), gene names/synonyms, one or more literature references and external hyperlinks to other online databases. Biomarker performance data, such as ROC curves, sensitivity, specificity, significance (*P*-value), etc. are available for >90% of the DNA biomarkers. Disease associated mutations were assumed to have 100% sensitivity/specificity, unless otherwise stated in the original source data. Details regarding how the SNP/GWAS biomarker performance values were calculated are provided in detail, elsewhere (10). Performance data for other DNA biomarkers were directly taken from the original literature sources and cited accordingly.

### Karyotype biomarkers

Karyotyping is a widely used practice of microscopically assessing chromosomal abnormalities to detect genetic disorders and classify cancers. Karyotype biomarkers (also called karyograms or idiograms) are sometimes categorized as imaging biomarkers. However, as karyotyping provides structural information about molecular (DNA) structure that is not readily available via sequencing, we chose to classify karyotype biomarkers as a special set of molecular biomarkers for MarkerDB. MarkerDB has a total of 154 karyotype markers or karyograms associated with 47 different diseases/conditions. All of these karyotype biomarkers are clinically approved for diagnostic, predictive or prognostic applications. All karyotype biomarker data in MarkerDB was extracted from the primary literature, including the Atlas of Genetics and Cytogenetics in Oncology and Haematology (18) and the Catalogue of Unbalanced Chromosome Aberrations in Man (19). Each karyotype idiogram was schematized and redrawn with the CyDAS software (20). By generating schematized idiograms/karyograms for most known disease-associated karyotypes it is hoped that the data MarkerDB would be compatible with interpreting both conventional karyotypes and virtual karyotypes or comparative genomic hybridization (CGH) data. The annotation of all karyotype biomarkers was done manually. Currently, all karyotype markers in MarkerDB have one or more disease associations along with a MarkerDB accession number, a labeled idiogram or karyogram (showing the normal karyotype adjacent to the diseased karyotype), a short description of the karyotype, the disease association, one or more literature references and hyperlinks to the associated genes. Biomarker performance data is generally not available for disease-associated karyograms or karyotypes.

## MarkerDB LAYOUT AND NAVIGATION

**Figure 1.**
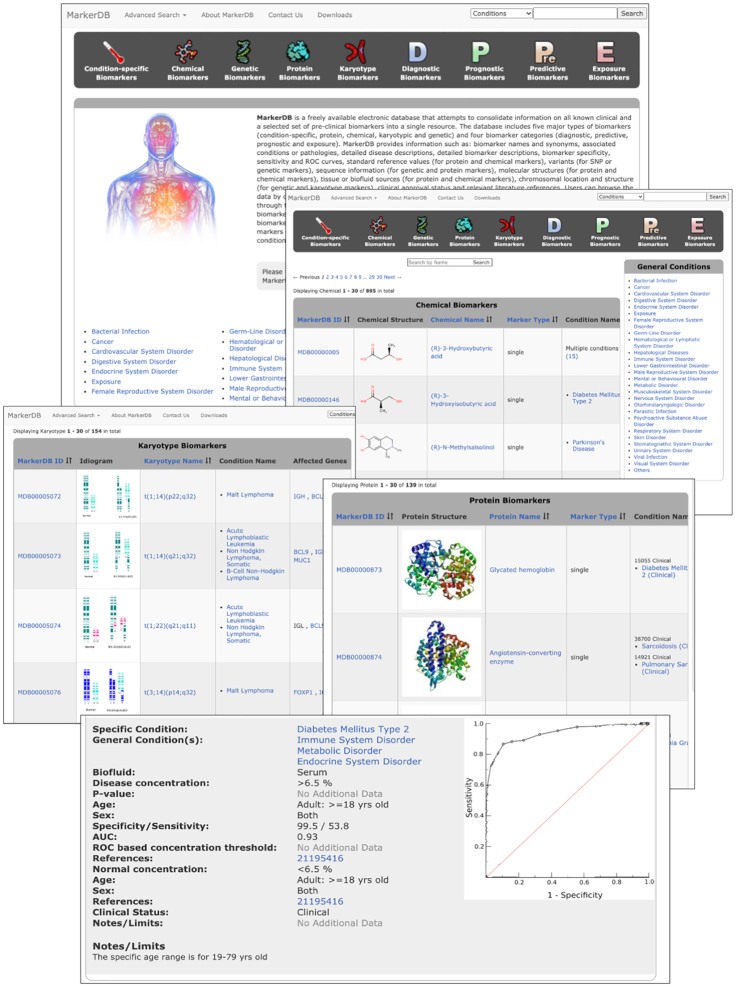
A screenshot montage of the MarkerDB interface. This figure shows five different views of the MarkerDB interface including the home page, the chemical biomarker view, the karyotype marker view, the protein marker view and the condition/disease view for a specific biomarker.

A screenshot montage of MarkerDB’s graphical user interface is shown in Figure 1. The interface is designed to allow users to easily browse, search and explore the full content of the database. A dark grey ‘Navigation Panel’ dominates the top portion of the home page. Nine pictographic icons, corresponding to three general biomarker browsing options within MarkerDB. These three general biomarker browsing options include: (i) browse biomarkers by disease, (ii) browse biomarkers by molecular type and (iii) browse biomarkers by purpose. Clicking on the ‘thermometer’ image or ‘Condition-specific Biomarkers’ allows users to view MarkerDB’s collection of biomarkers listed by condition or disease. The resulting three-column table displays the specific conditions or diseases alphabetically (column 1), the associated biomarkers (column 2) and the general disease/condition category (column 3). Each page in the ‘Condition-specific Biomarker’ table shows 30 entries, which can be browsed page-by-page by clicking the hyperlinked page numbers at the top or bottom of the table. The ‘Condition Names’ in column 1 can be re-sorted (alphabetical or reverse alphabetical order) by clicking the up/down arrows beside the column title. There is a text search box at the top of the page, that allows uses to search the ‘Condition-specific Biomarker’ table by specific condition names. This is done by typing names or partial names into the search box and pressing the ‘Search’ button on the right. All entries in the ‘Condition-specific Biomarker’ table are hyperlinked. Clicking the name of a condition (in column 1) will generate a new table view that displays a short description of the condition along with the known biomarkers (grouped according to their molecular categories) and details regarding the biomarker performance, reference values, literature references and applicability. The ‘Biomarker’ column (column 2) displays all known biomarkers for a given condition along with the MarkerDB accession number (MDB-number), molecular category and biomarker class to which these markers belong. Clicking on the name of the biomarker will generate a new table view that displays a short description of the biomarker along with the known conditions or diseases associated with that biomarker along with details regarding the biomarker's performance, reference values, literature references and applicability. The ‘Condition Categories’ column (column 3) displays the general conditions that a specific disease or conditions is associated with. Clicking on the name or names in this column will generate a new table that provides a definition of the condition category along with a list of all diseases/conditions in MarkerDB that are associated with that condition category. The ‘General Condition Categories’ can also be accessed by clicking on the small table of 27 General Conditions found on the right side of most table views seen in MarkerDB or at the bottom of the MarkerDB homepage.

Returning to the Navigation panel at the top of the MarkerDB homepage, users can also browse biomarkers by molecular type (option 2). Clicking on either the ‘Chemical Biomarkers’ (with a chemical pictogram), ‘Genetic Biomarkers’ (with a DNA pictogram), ‘Protein Biomarkers’ (with a protein pictogram) or ‘Karyotype Biomarkers’ (with a chromosome pictogram) allows users to view biomarkers listed by their molecular categories. Clicking the ‘Chemical Biomarkers’ icon generates a browsable and sortable table consisting of five columns, including the MarkerDB ID, the chemical structure, the chemical name, the marker type (singular or a multi-marker panel) and the specific conditions associated with that chemical biomarker. Clicking on the hyperlinks within the chemical biomarker table will take users to additional tables with more details about the compound(s), the biomarker performance or the disease(s). Clicking the ‘Genetic Biomarkers’ icon generates a browsable and sortable table consisting of six columns, including the MarkerDB ID, the marker type, the gene name, the chromosome number (if known), the chromosome position (if known) and the specific conditions associated with that genetic biomarker. At the top of the table a filter selector is available to filter the list of biomarkers by chromosome number or bacterial/viral categories. Clicking on the hyperlinks within the genetic biomarker table will take users to additional tables with more details about the gene(s), the variants or mutations, the sequence(s), the biomarker performance and/or the disease(s). Selecting the ‘Protein Biomarkers’ icon generates a browsable and sortable table consisting of four columns, including the MarkerDB ID, the protein structure, the protein name, and the specific conditions associated with that protein biomarker. Clicking on the hyperlinks within the protein biomarker table will take users to additional tables with more details about the protein(s), the sequence(s), the biomarker performance and/or the disease(s). Clicking on the ‘Karyotype Biomarkers’ icon generates a browsable and sortable table consisting of five columns, including the MarkerDB ID, the ideogram or karyogram, the karyotype name, the specific conditions associated with that karyotype biomarker and the affected gene(s). Clicking on the hyperlinks within the protein biomarker table will take users to additional tables with more details about the protein(s), the sequence(s), the biomarker performance and/or the disease(s).

Once again returning to the Navigation panel at the top of the MarkerDB homepage, users can also browse biomarkers by biomarker purpose (option 3). That is, users can choose to browse only ‘Diagnostic Biomarkers’ (with a D), ‘Prognostic Biomarkers’ (with a P), ‘Predictive Biomarkers’ (with a Pre) or ‘Exposure Biomarkers’ (with an E). Clicking on any of the four icons will generate a browsable, sortable table consisting of four columns, including the MarkerDB ID, the biomarker structure (if available), the biomarker name, and the specific conditions associated with that biomarker. At the top of each table is a filter selector that allows users to filter the list of biomarkers by molecular category (protein, gene, chemical, karyotype). Clicking on the hyperlinks within any of the four biomarker purpose tables will take users to additional tables with more details about the biomarker(s), ideogram(s), structure(s), sequence(s), biomarker performance and/or the disease(s). More details about navigating and browsing MarkerDB are available through its online manual, which is located under the ‘About MarkerDB’ menu tab.

In addition to the many browsing and display filtering options already described, MarkerDB also provides a number of searching utilities. On the upper right-hand corner, a text search box is available to allow users to search the entire website or to limit the search to specific fields, such as conditions, all markers, chemical biomarkers, genetic biomarkers, etc. As with the other text search utilities, an auto-filling feature is provided to help facilitate the search and perform spelling corrections. MarkerDB also supports advanced text searches (>20 different data field categories), sequence (DNA and protein) searches and structure (chemical) searches through the ‘Advanced Search’ option available at the top of the home page. The sequence search uses BLASTN for DNA sequence searches and BLASTP for protein sequence searches (21). All returned sequence matches are hyperlinked to the relevant biomarker page. The chemical structure search utility uses the MarvinView Applet from ChemAxon, which allows users to interactively draw structures (or paste InChI or SMILES strings) into the drawing pallet and to search for similar structures using the Tamimoto similarity index.

The MarkerDB website was designed to take advantage of a number of recent improvements to web-based tools, frameworks and caching systems to make the website more user friendly and responsive. The design is inspired by the Twitter Bootstrap framework, which makes for much easier navigation and a more appealing user experience. Like other databases developed by our group, MarkerDB server uses Redis-based caching that makes the loading of data, structures, images and sequences very fast. To facilitate rapid prototyping and development, the entire MarkerDB database has been built upon an MVC (Model-View-Controller) framework called Ruby on Rails. In the MVC framework, models respond and interact with the data by connecting to the database, views create the interface to show and interact with the data, and controllers connect the user to the views. Such a framework allowed our database developers to easily create code for each of the respective modules in MarkerDB. This framework is particularly robust and code can be reused in different functions or changed easily to accommodate future plans or abrupt changes in design. In particular, this allowed our development team to liberally borrow code and functions from other databases developed in our lab (8,22–24).

## MarkerDB ASSEMBLY, QUALITY CONTROL, CURATION AND FAIRness

MarkerDB was assembled with the same quality assurance, quality control and data compilation procedures implemented for many of the databases developed by our group, including HMDB (8), DrugBank (22), YMDB (23) and ECMDB (24) In particular, all biomarkers in MarkerDB were identified using a combination of manual literature surveys, text mining of on-line journals or textbooks and data mining of various electronic databases. The exact journals, textbooks and online data sources for many specific biomarker classes have already been described earlier and are provided in MarkerDB’s references. To ensure completeness, additional checks for common biomarkers (and biomarker tests) were conducted by studying commercial, government lab or hospital laboratory test lists from across North America (many of which are listed on line). In order to ensure both completeness and correctness, each biomarker record entered into MarkerDB was reviewed and validated by a member of the curation team after being annotated by another member. Other members of the curation group routinely performed additional spot checks on each entry.

Several locally developed software packages including text-mining tools, physico-chemical parameter calculators, as well as chemical, gene and protein annotation tools (DataWrangler, ChemoSummarizer, BioSummarizer) were modified specifically for MarkerDB and used to facilitate data entry and data validation. To monitor the data entry process, all of MarkerDB’s data is entered into a centralized, password-controlled database, allowing all changes and edits to MarkerDB to be monitored, time-stamped and automatically transferred. All members of the MarkerDB curation team were required to have at least an undergraduate degree in bioinformatics or molecular biology. This ensured that they had sufficient biological and/or biochemical knowledge to understand and interpret the scientific literature, the disease or conditions and the performance parameters associated with the biomarker data. All curation team members were also given extensive training by the lead curator(s) in biomarker annotation via hands-on mentoring, text instructions, peer support, and tutorials.

Improvements and updates to MarkerDB’s content are an ongoing process. Minor corrections or small additions to a biomarker entry or its layout will be done without a formal update announcement. However, significant changes, additions, or improvements to an individual biomarker entry will be listed in the MarkerDB ‘marker-card’ and the last update date will be modified to reflect any such changes. As this is only version 1.0 of MarkerDB, all biomarker entries are dated with August 2020 as the last update date. Large-scale updates and improvements to the database in the future will be given database version numbers (2.0, 3.0, etc.) and suitable database update dates. They will also be described in detail as publications or online update descriptions as appropriate.

MarkerDB is FAIR compliant (3) and details regarding its ‘FAIRness’ are provided under the ‘About MarkerDB’ menu tab. To ensure findability, all marker entries in MarkerDB have a unique and permanent 7-digit MDB identifier. To ensure accessibility, MarkerDB not only provides a well-supported web-based user-interface with extensive search functions, it also provides an application programming interface (API) located under the ‘About MarkerDB’ menu tab, to support programmatic access to the data. To ensure interoperability, all diseases or conditions are mapped to established ontologies (Disease Ontology, SNOMED CT and ICD-10 [International Classification of Diseases, version 10]) and all molecular data have clear references to other established reference, meta-data or data resources. An extensive and well-annotated data download section is also provided with files available in standard *.tsv and XML formats. To ensure re-usability all of the data in MarkerDB is extensively sourced with clear provenance. The data in MarkerDB are released under a Creative Commons Attribution BY and NC license.

## MarkerDB LIMITATIONS AND FUTURE PLANS

As has been noted earlier, MarkerDB is a database focused on molecular, biomedical biomarkers. It is not a database containing imaging biomarkers, cellular/tissue biomarkers or non-medical (i.e., agricultural, aquaculture, wildlife, botanical, etc.) biomarkers. This means that MarkerDB does not contain histological, flow cytometry or tissue biomarkers, nor does it include X-ray, CT, PET, MRI imaging biomarkers derived from other medical imaging modalities. Given the difficulty of compiling, displaying and categorizing these types of markers, it is unlikely that MarkerDB will ever include them in the future. Our focus on molecular biomarkers for MarkerDB was quite deliberate as we believe these markers can be more objectively measured, more easily displayed and are more compatible with an online database. While considerable effort has been made by the MarkerDB curation team to capture well-known, widely used and clinically approved biomarkers, there are no doubt some biomarkers or newly emerging molecular biomarker types that have been missed. In particular, we are aware that MarkerDB currently does not contain microRNA (miRNA) biomarkers, methylation (DNA) markers nor transcript (mRNA) biomarkers. The relatively small number of clinically approved biomarkers in these categories was the primary reason for leaving them out of version 1.0 of MarkerDB. However, given the exciting findings and rapid developments occurring with these kinds of biomarkers, we expect that they will be included in version 2.0. Likewise, MarkerDB currently does not include any therapeutic, adverse drug effect or drug efficacy biomarkers. This is another area that we are actively exploring and we expect that these markers will be included in version 2.0 of the database.

Users of MarkerDB may also note the relatively modest number of pre-clinical or investigative biomarkers listed in the database. As has been noted earlier, the quality and content of most biomarker research papers precludes their inclusion of most of their biomarker findings in MarkerDB. This is because they rarely provide the needed information on biomarker performance, cut-off thresholds, statistical significance or other measures of sensitivity or specificity that are required for FDA, EU (the European Medicines Agency or EMA) or LDT approval of most molecular biomarkers. Gratifyingly there is a trend towards improved quality and rigor in more recent biomarker papers, so it is likely that more of the data from these investigative reports could be included in the next release of MarkerDB.

As with most databases, MarkerDB is a work in progress. Indeed, the number and type of biomedical biomarkers is constantly evolving and expanding. This is due to the ongoing development and approval of new commercial biomarker tests, new EMA or FDA-approved tests, new LDTs, new technologies, the appearance of new diseases (COVID-19), and the constant flow of newly published or patented research on biomarkers. As has been remarked earlier, the published literature on biomarkers is vast (65 000 papers/year) and so without some kind of quality filter it is impossible to stay current with all published investigational biomarkers. However, by focusing on those papers with very high quality, quantitative data and other rich biomarker performance data (i.e. sensitivity, specificity, ROC curve, reproducibility, statistical significance, and the availability of threshold or cut-off values), we believe that the number of papers needing the attention of MarkerDB’s curators could be kept to a manageable number.

Overall, we are aware of MarkerDB’s shortcomings and have plans to address these in future releases. However, we are also looking forward to receiving feedback for version 1.0 of MarkerDB from the user community as this often opens our eyes to other, less obvious shortcomings in the database's design, layout, logical flow and content. As always, we hope to be responsive to these comments and to engage the user community to make MarkerDB and its subsequent updates as useful, informative and reliable as possible.

## CONCLUSION

MarkerDB represents a new type of open access, online biomarker database that brings many elements of modern web design and modern web-based databasing to the biomarker field. More importantly, MarkerDB provides a level of coverage and depth that has not been seen in other publicly accessible biomarker databases. In particular, MarkerDB provides the broadest coverage of the greatest diversity of molecular biomarkers (chemical, protein, genetic, karyotypic) for the widest number of biomarker purposes (diagnostic, prognostic, predictive and exposure) and the broadest range of diseases and conditions. In addition to providing broad, in-depth biomarker coverage, a major focus of MarkerDB is in the provision of detailed information, not only about the biomarker itself, but also about its performance, its approval status and the disease or condition with which the biomarker is associated. By creating a user-friendly, comprehensive, richly annotated biomarker database of medically relevant molecular biomarkers we hope to help advance the field of biomarker research and biomarker use. In particular, we hope this first version and future updates to MarkerDB will provide biologists, biochemists, geneticists, biomedical researchers, clinicians and patients with readily accessible information that can be used to inform them about the status, quality and utility of different biomarkers for different conditions. We also believe MarkerDB will allow scientists and clinicians to determine what molecular biomarkers are known for a given disease, what biomarkers are approved for clinical use, which biomarkers or biomarker tests are most useful, or whether it is even worthwhile to pursue finding a new biomarker.
